# Islands Spark Accelerated Evolution

**DOI:** 10.1371/journal.pbio.0040334

**Published:** 2006-09-12

**Authors:** Liza Gross

The notion of islands as natural testbeds for evolutionary study is nearly as old as the theory of evolution itself. The restricted scale, isolation, and sharp boundaries of islands create unique selective pressures, often to dramatic effect. Following what’s known as the “island rule,” small animals evolve into outsize versions of their continental counterparts while large animals shrink. Once restricted to islands, small animals often lacked predators and the competition between species that constrained the growth of their relatives on the mainland. Large mammals, on the other hand, no longer had access to vast grasslands and other abundant food sources and grew smaller to survive. Giant tortoises and iguanas still inhabit the Galápagos and a few other remote islands today, but only fossils remain of the dwarf hippopotami, elephants, and deer that once lived on islands in Indonesia, the Mediterranean, and the Pacific Ocean.

The fossil record suggests that these size changes (as well as other morphological changes) occur rapidly after species become isolated on islands, but this assumption has never been empirically examined in a systematic manner. In a new study, Virginie Millien puts this longstanding hypothesis to the test by analyzing the fossil record and data from living species. Comparing the rates of evolutionary change between island and mainland populations for 88 species at intervals ranging from 21 years to 12 million years, Millien confirmed that island species undergo accelerated evolutionary changes over relatively short time frames, between decades and several thousand years. (One can imagine rats of horror movie proportions if such rates persisted for millions of years.)

Measuring rates of evolutionary change has proven difficult because the fossil record rarely captures every morphological shift in a lineage, precise dating isn’t always possible, and it’s often not clear when the ancestral form first appeared on the island. To get a robust sample of island and mainland mammalian species, Millien collected data from text, figures, and tables in an extensive survey of the published literature. From these datasets, she calculated a total of 826 evolutionary rates for 170 populations representing the 88 species. (Rates of evolutionary change are measured in units called, appropriately enough, “darwins.”)

Evolution rates, she found, decreased over time intervals for both island and mainland species, with a slower rate of decrease for island species. The differences in evolutionary rates between island and mainland pairs also decreased over time, becoming statistically insignificant for intervals over 45,000 years. Overall, island species evolved faster than mainland species—a phenomenon that was most pronounced for intervals between 21 years through 20,000 years.

Island evolution theory predicts that the most extreme effects of isolation will be seen on the smallest, most far-flung islands. In keeping with theory, Millien found that evolutionary rates for different populations of the same species varied with island locale. Thus, the rate of evolution does not appear to be an evolutionarily conserved trait, like metabolic rate or whiskers.

**Figure:**
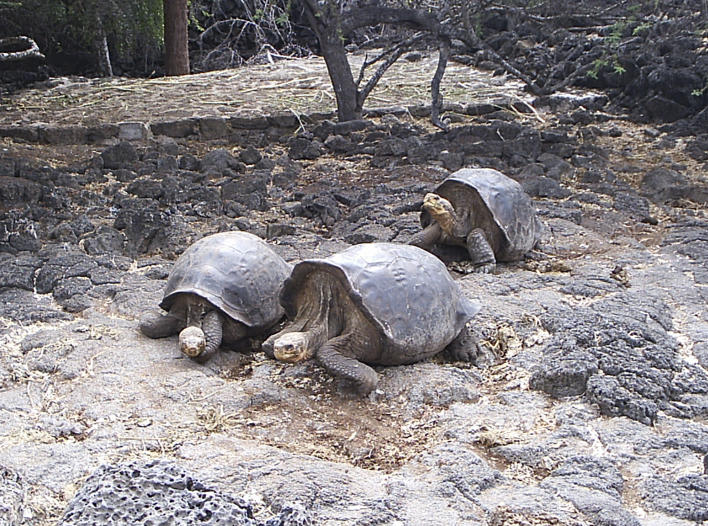
Giant tortoises at the Darwin Station on Isla Santa Cruz in the Galápagos Islands. (Photo: Catriona MacCallum)

Because rodents make up nearly half of the world’s mammalian species—and over 70% of taxa on some islands in this study—Millien repeated her analysis on a subset of the data with equal numbers of rodent and non-rodent taxa. The overrepresentation of rodents had no effect on the results, which still revealed significant differences between island and mainland evolution rates for the same species or populations.

The finding that mammals evolve faster on islands, Millien argues, comports with the island evolution theory prediction that mammals respond to their new island homes with rapid morphological and size adaptations. The brisk pace of these changes may explain why the fossil record harbors few examples of intermediate forms between the mainland ancestor and island descendant. Millien’s results also conform with the hypothesis that evolution rates for island species slow down after the initial period of accelerated change, approaching rates on the mainland.

If island species can evolve quickly, Millien argues, it stands to reason that mainland species retain a similar capacity. As habitat destruction continues to pose the number one threat to biodiversity, many mainland habitats are beginning to resemble islands, with isolated pockets of wildlife separated by degraded or developed lands. Thus, island species may serve as a model for understanding how mainland species will adapt to the rapidly changing environmental conditions brought on by habitat destruction and global warming. It appears that some mainland species are already responding like island species: a 1989 study followed the island rule in linking fragmented habitat to body size changes in 25 European mammals over the past 200 years. How long animals can continue to evolve in the face of these changes, however, remains to be seen.

